# A Novel Fungal Metabolite with Beneficial Properties for Agricultural Applications

**DOI:** 10.3390/molecules19079760

**Published:** 2014-07-08

**Authors:** Francesco Vinale, Gelsomina Manganiello, Marco Nigro, Pierluigi Mazzei, Alessandro Piccolo, Alberto Pascale, Michelina Ruocco, Roberta Marra, Nadia Lombardi, Stefania Lanzuise, Rosaria Varlese, Pierpaolo Cavallo, Matteo Lorito, Sheridan L. Woo

**Affiliations:** 1 Istituto per la Protezione Sostenibile delle Piante, Consiglio Nazionale delle Ricerche, via Università 133, 80055 Portici, Italy; 2 Dipartimento di Agraria, Università di Napoli ‘Federico II’, Portici, 80055 Naples, Italy; 3 Centro Interdipartimentale di Spettroscopia di Risonanza Magnetica Nucleare (CERMANU), Università degli Studi di Napoli ‘Federico II’, Portici, 80055 Naples, Italy; 4 Dipartimento di Fisica, Università di Salerno, Via Giovanni Paolo II, 132, 84084 Fisciano, Italy

**Keywords:** *Trichoderma*, secondary metabolites, isoharzianic acid, harzianic acid, plant growth promotion, disease resistance

## Abstract

*Trichoderma* are ubiquitous soil fungi that include species widely used as biocontrol agents in agriculture. Many isolates are known to secrete several secondary metabolites with different biological activities towards plants and other microbes. Harzianic acid (HA) is a *T. harzianum* metabolite able to promote plant growth and strongly bind iron. In this work, we isolated from the culture filtrate of a *T. harzianum* strain a new metabolite, named isoharzianic acid (iso-HA), a stereoisomer of HA. The structure and absolute configuration of this compound has been determined by spectroscopic methods, including UV-Vis, MS, 1D and 2D NMR analyses. *In vitro* applications of iso-HA inhibited the mycelium radial growth of *Sclerotinia sclerotiorum* and *Rhizoctonia solani*. Moreover, iso HA improved the germination of tomato seeds and induced disease resistance. HPLC-DAD experiments showed that the production of HA and iso HA was affected by the presence of plant tissue in the liquid medium. In particular, tomato tissue elicited the production of HA but negatively modulated the biosynthesis of its analogue iso-HA, suggesting that different forms of the same *Trichoderma* secondary metabolite have specific roles in the molecular mechanism regulating the *Trichoderma* plant interaction.

## 1. Introduction

The use of microbes for pest management in agriculture is one of the most effective biological control strategies. The beneficial effects are strain dependent and the advantages for the associated plant include the suppression of pathogens by using a variety of mechanisms (*i.e.*, antibiosis, parasitism, competition for nutrients, *etc.*), the promotion of plant growth and the improvement of host resistance to both biotic and abiotic stresses [1,2,3].

Secondary metabolites are chemically different natural compounds of relatively low molecular weight (in most cases < 3 kDa), that are mainly produced by microorganisms and plants, and typically associated to individual genera, species or strains. They are biosynthesized along specialized pathways from primary metabolites, exhibit a wide range of biological activities and play an important role in regulating interactions between organisms [4]. Included in this group are antibiotics, which are natural products capable of inhibiting or killing microbial competitors [5,6].

In fungi, the production of secondary metabolites has been often correlated to specific stages of morphological differentiation, and associated to the phase of active growth [7]. Interestingly, some fungal secondary metabolites can modify the growth and the metabolism of plants, while others seem to target specific fungal processes such as sporulation and hyphal elongation [7]. Thus, the expression of secondary metabolites may occur at a predictable point during the normal life cycle of some fungi, including those used for agriculture applications [7].

Some fungi of the genus *Trichoderma* may act as symbionts of plants, and are presently marketed as biopesticides and biofertilizers due to their ability to protect crops and promote vegetative growth [1,2,3]. These microbes are well known producers of secondary metabolites with different biological activities [8,9,10]. The production of such compounds varies according to the strain and in relation to the equilibrium between elicited biosynthesis and biotransformation rates (or degradation by other microbes) [11]. 

In this work we report the isolation and the characterization of a new metabolite named isoharzianic acid (iso-HA), a stereoisomer of harzianic acid, from the culture filtrate of a *T. harzianum* strain isolated from decomposing hardwood bark. The biological activity of this metabolite was investigated both *in vitro* against the fungal pathogens *Sclerotinia sclerotiorum* and *Rhizoctonia solani* and *in vivo* in terms of plant growth promotion and induction of disease resistance. Moreover, the influence of plant tissue on the production of HA and iso-HA has been also examined.

## 2. Results and Discussion

**Figure 1:**
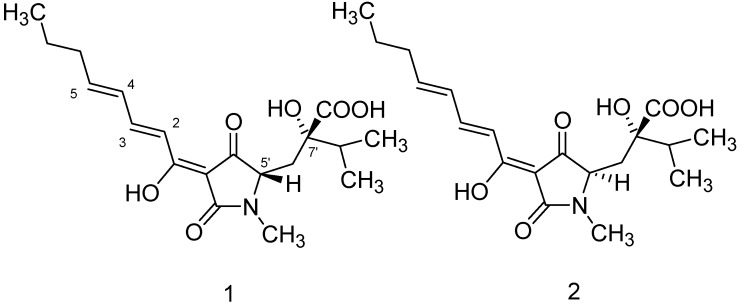
Chemical structures of (**1**) HA; (**2**) iso-HA.

**Figure 2:**
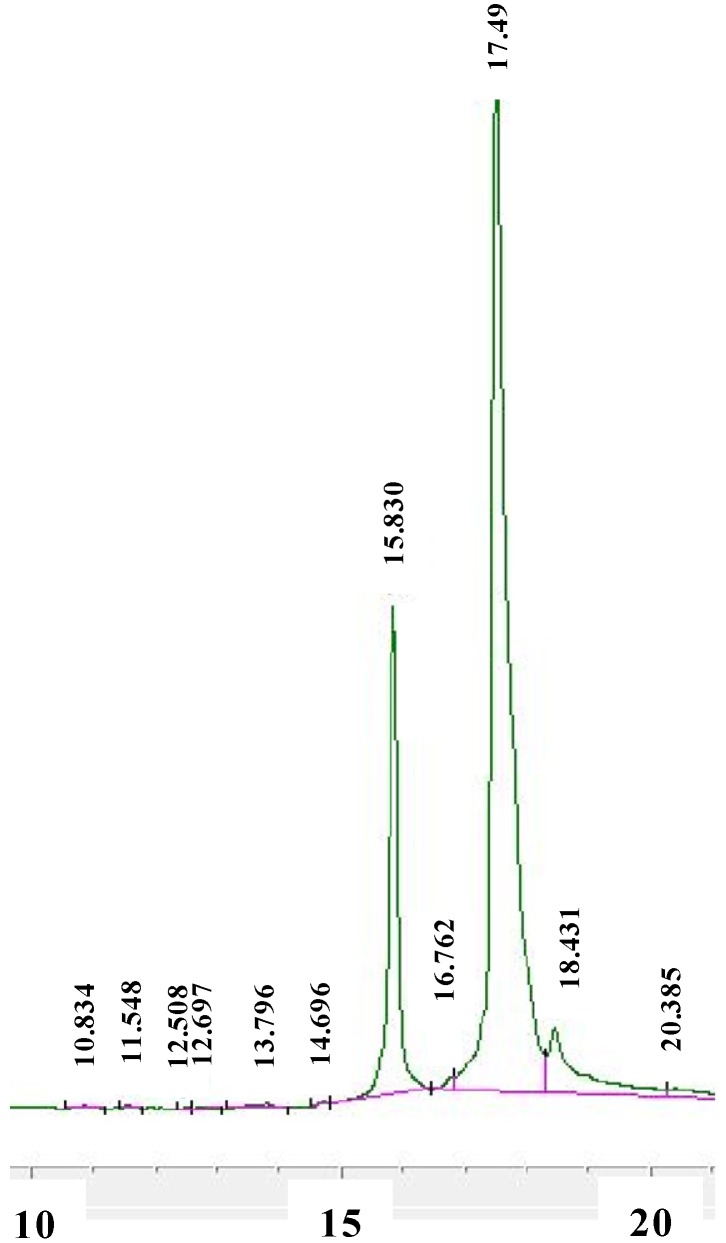
Chromatogram of *T. harzianum* extract, as monitored by HPLC-DAD at 360 nm (10 to 20 min).

*T.* *harzianum* culture filtrate was extracted exhaustively with ethyl acetate to give a red-brown residue from which HA (**1**) and iso-HA (**2**) (Figure 1) were isolated after RP-18 vacuum chromatography or semi-preparative HPLC (Figure 2). The structure of **1** was determined by comparison of its NMR spectroscopic data with those of an authentic standard [12,13]. The absolute configuration of **1**, determined by X-ray diffraction studies, its antibiotic activity and plant growth promotion effect have been reported in a previous study [13]. Recently, we demonstrated the ability of this tetramic acid to bind with a good affinity essential metals such as Fe^3+^, thus representing a previously unrecognized siderophore [14].

iso-HA was obtained as a yellow solid and its ESI-MS/MS spectrum showed a molecular ion peak at 366.1909 *m/z* corresponding to C_19_H_27_NO_6_. HA (**1**) and iso-HA (**2**) had similar mass spectra with molecular ions at *m/z* 366 ([M+H]^+^) and main fragments at *m/z* 320 ([M+H-HCO_2_H]^+^), 224 and 138. Interestingly, HA showed an extra peak at 348 *m/z* ([M+H-H_2_O]^+^). The optical rotation of iso-HA is [α]_D_ −15 (c 1.1, MeOH),while is [α]_D_ +16 (c 1.06, MeOH) for HA [12]. 

The ^1^H- and ^13^C-NMR spectra of these two compounds (Table 1) showed high similarities. The analyses of mono- and bidimensional NMR spectra showed that these two metabolites have the same signals except H-5' and H-6', suggesting a different stereochemistry of C5 and C6. NOESY experiments were performed to confirm this hypothesis and allowed to determine the configuration of the two stereoisomers. In particular, NOESY experiments revealed, in case of HA, a through-space correlation between the H-5' and H-6' b protons, which, conversely, was not detected in case of iso-HA. However, no other different correlations between HA and iso-HA were observed, thus suggesting the different orientation of H-5'.

**Table 1:** ^1^H- and ^13^C-NMR spectral data of HA and iso-HA (in CD_3_OD).

Position	HA (1)				iso-HA (2)			
	δ ^13^C	δ ^1^H	Multi	J (Hz)	δ ^13^C	δ ^1^H	Multi	J (Hz)
1	174.0	_		_	174.0	_		_
2	119.3	7.0	d	15.6	119.4	7.05	d	15.25
3	146.2	7.57	m	_	146.2	7.47	dd	10.17; 5.4
4	129.7	6.35	m	_	129.7	6.35	m	_
5	148.5	6.30	m	_	148.5	6.30	m	_
6	35.4	2.19	m	_	35.4	2.19	m	_
7	21.8	1.50	m	_	21.8	1.50	m	_
8	13.7	0.93	m	_	13.7	0.93	m	_
2'	174.0	_		_	174.0	_		_
3'	99.6	_	q	_	99.5	_	q	_
4'	195.0	_		_	195.1	_		_
5'	63.7	3.62	dd	1.17, 9.3	63.6	3.80	dd	2.7, 4.6
6'a	34.9	2.20	dd		34.8	2.2	c	
6'b		2.51				2.2		
7'	78.1	_	q	_	78.0	_	q	_
8'	35.9	2.02	m	_	35.8	2.02	m	_
9'	17.2	0.98	m	_	17.2	0.98	m	_
10'	16.4	0.99	m	_	16.4	0.99	m	_
11'	27.4	2.99	s	_	27.3	2.98	s	_
12'	176.8	_		_	176.7	_		_

c: Overlapping NMR signals. Abbreviation, s: singlet, d: doublet, dd: doublet of doublets, m: multiplet, q: quartet.

All these data implies that the metabolite 2-hydroxy-2-[4-(1-hydroxyocta-2,4-dienylidene)-1-methyl-3,5-dioxopyrrolidin-2-ylmethyl]-3-methylbutyric acid named iso-HA is a diastereoisomer of HA with the stereochemistry reported in Figure 1. The difference between these two tetramic acids determines their different chemical and physical characteristics (UV λ_max_ nm (log ε) HA = 344 (3.11), iso HA = 340 (4.04), different solubility in organic solvents).

**Figure 3:**
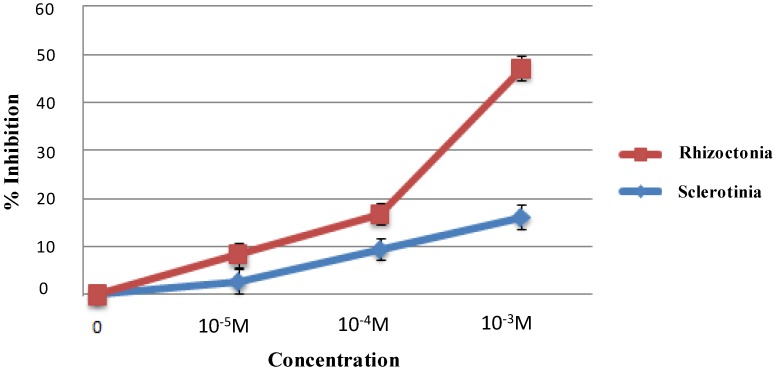
Antibiotic activity of iso-HA at different concentrations on *Rhizoctonia solani* (Rhizoctonia -■-) and *Sclerotinia sclerotiorum* (Scletotinia -♦-). % Inhibition of radial growth.

*In vitro* assays were performed to assess the iso-HA antibiotic activity. This compound, at concentration of 10^−3^ M, inhibited the growth of the phytopathogenic agents *R. solani* and *S. sclerotiorum* of about 40% and 20%, respectively, while at concentrations of 10^−4^ M and 10^−5^ M it showed lower effects compared to untreated control (Figure 3). No significant growth inhibitions were observed with other two fungal pathogens, *Botrytis cinerea* and *Phytium ultimum* (data not shown).

**Figure 4:**
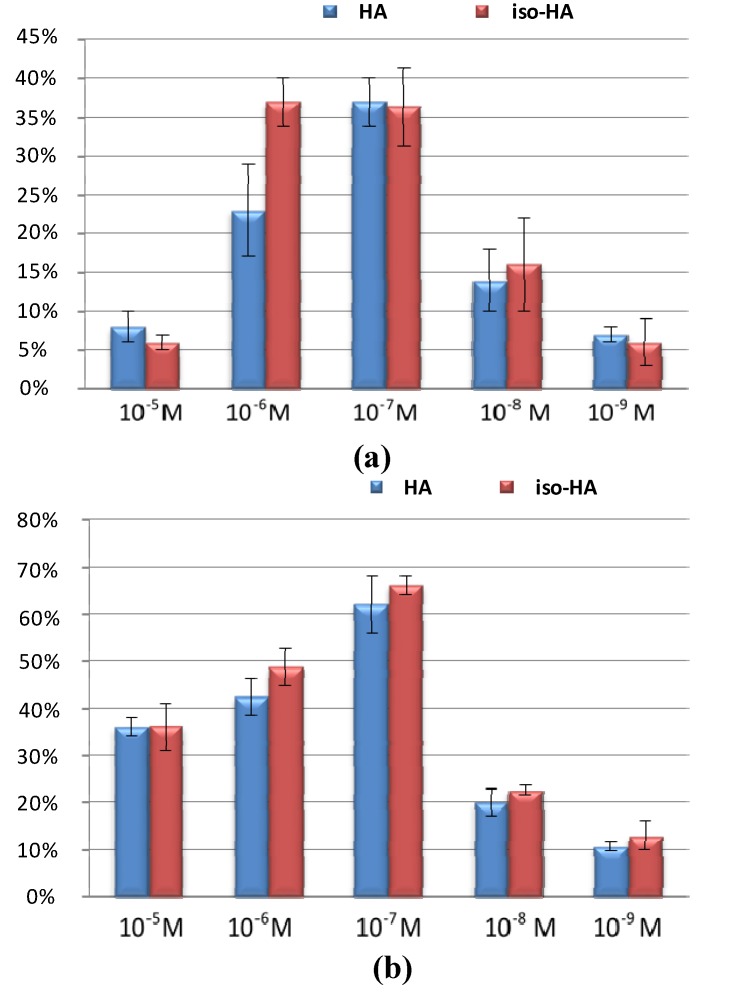
*In vitro* plant growth promotion effects of iso-HA (10^−5^, 10^−6^, 10^−7^, 10^−8^ and 10^−9^ M) on tomato seedlings. (**a**) Stem and (**b**) root lengths. Values indicates the % increase of growth as compared to untreated control plants. Each bar is the mean ± the standard deviation.

The *in vitro* effect of iso-HA and HA on tomato growth was evaluated in terms of seed germination, stem and root lengths (Figure 4). Both metabolites promoted seed germination (Table 2) and plant growth, with an increase of 35% in stem length (iso-HA and HA 10^−7^ M) and of 65% in root length (iso-HA and HA 10^−7^ M). Interestingly, iso-HA enhanced the plant fresh weight at 10^−6^ and 10^−7^ M, while small differences were observed with HA treatments.

**Table 2:** *In vitro* effect of iso-HA and HA on tomato seed germination (12, 24, 36, 48 h after sowing). DS = standard deviation.

% of Germination								
Treatment	12 h	DS	24 h	DS	36 h	DS	48 h	DS
Control	0%	0%	0%	0%	55%	3.6%	100%	0%
HA 10^−5^ M	0%	0%	50%	3.9%	100%	0%	100%	0%
HA 10^−6^ M	0%	0%	72%	4.1%	88%	3.4%	100%	0%
HA 10^−7^ M	0%	0%	55%	3.1%	100%	0%	100%	0%
HA 10^−8^ M	0%	0%	61%	7.3%	76%	12.3%	100%	0%
HA 10^−9^ M	0%	0%	56%	7.9%	63%	11.1%	100%	0%
iso-HA 10^−5^ M	0%	0%	72%	3.9%	100%	0%	100%	0%
iso-HA 10^−6^ M	0%	0%	66%	5.2%	100%	0%	100%	0%
iso-HA 10^−7^ M	0%	0%	88%	5.6%	100%	0%	100%	0%
iso-HA 10^−8^ M	0%	0%	62%	2.6%	83%	11.8%	100%	0%
iso-HA 10^−9^ M	0%	0%	56%	2.8%	67%	3.8%	100%	0%

**Figure 5:**
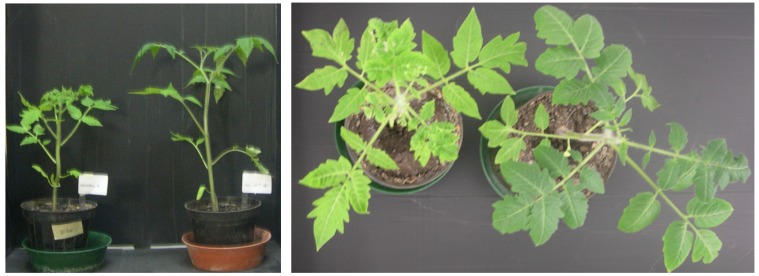
*In vivo* plant growth promotion effects of iso-HA on tomato. (**Left**) untreated control; (**Right**) plant treated with iso-HA 10^−6^ M.

**Figure 6:**
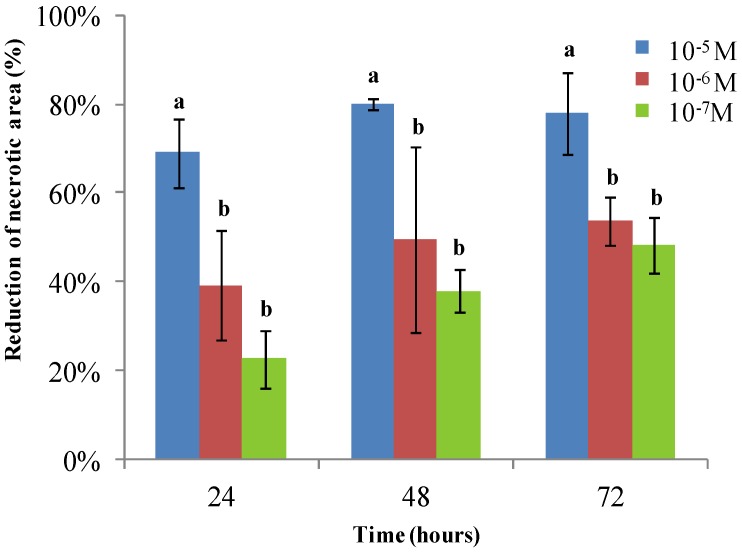
Induction of disease resistance against *B. cinerea*. Plants were drenched with iso-HA at different concentrations (10^−5^, 10^−6^, 10^−7^ M). Each bar is the mean ± the standard deviation. Treatments with the same letter are not significantly different (*p* < 0.05).

*In vivo* treatment of tomato plants with iso-HA increased stem length by 22%, 35% and 19% at concentrations of 10^−5^, 10^−6^, 10^−7^ M, respectively, compared to untreated control (Figure 5). Moreover, the ability of iso-HA to induce systemic resistance against *B. cinerea* was evaluated*.* A reduction of the necrotic area (90%) caused by the pathogen was observed 48 h after a drench application of iso-HA at 10^−5^ M (Figure 6).

Some *Trichoderma* strains produce compounds that can cause substantial changes in the metabolism of the host plant [10,15]. The involvement of secondary metabolites in the ability of *Trichoderma* spp. to activate plant defence mechanisms and regulate plant growth has been investigated [16,17]. HA is a natural product that demonstrates antifungal and plant growth promoting activities [13]. In this paper we indicate that iso-HA is an antifungal compound and also an inducer of plant disease resistance.

**Figure 7:**
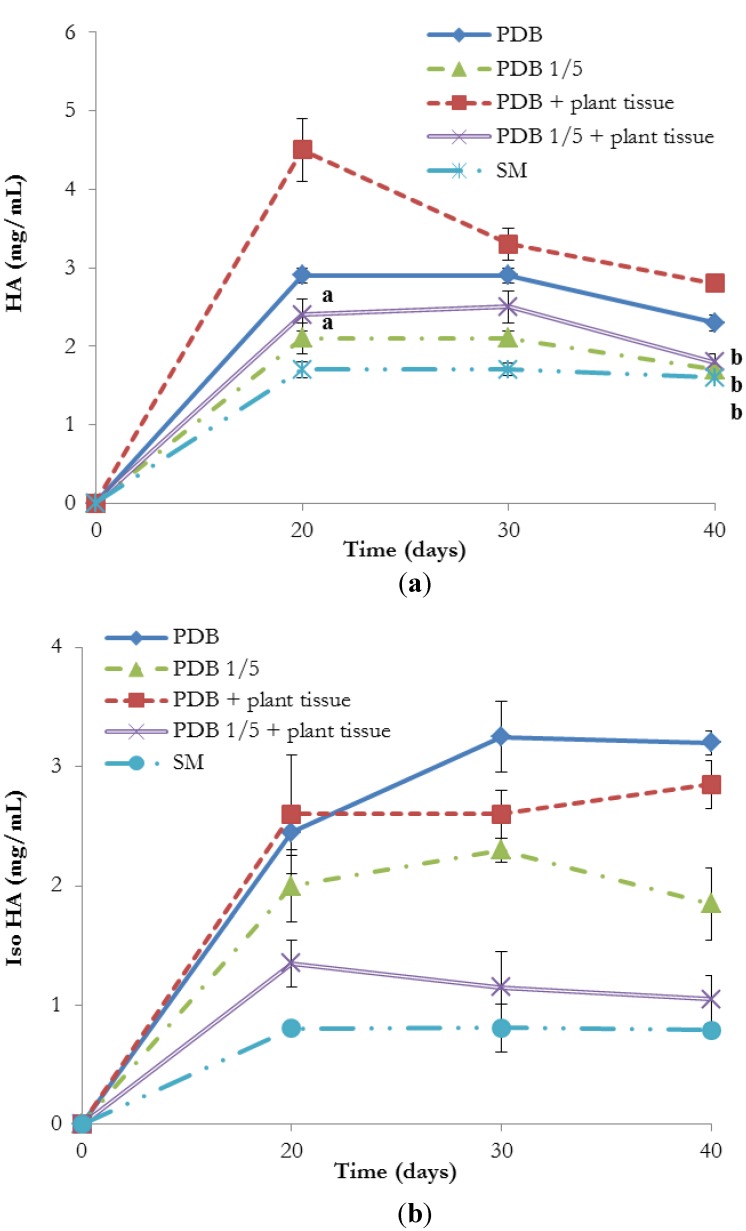
Production of HA (**a**) and iso-HA (**b**) in different media amended with tomato plant tissue. PDB = full PDB (-♦-); PDB 1/5 = 1/5 strength PDB (-▲-); PDB + plant tissue = full PDB amended with tomato plant tissue (-■-); PDB 1/5 + plant tissue = 1/5 strength PDB emended with tomato plant tissue (**-x-**); SM = salt medium with 1% glucose (-●-). Each point on the line is the mean ± the standard deviation of four independent biological replicates. Point on the line with the same letter are not significantly different; point on the line without the letter are significantly different (*p* < 0.05).

In order to test if the presence of plant may influence the production of HA and iso-HA in *T. harzianum*, tomato tissue was added to the cultivation media (PDB, PDB 1/5 strength and SM) in order to mimic the composition of a natural substrate or a naturally occurring plant-microbe interaction. The presence of tomato plant modulated the production of the tetramic acid derivatives as reported in Figure 7. For both metabolites, the production was significantly higher in potato dextrose broth (PDB—full and 1/5 strength) compared to the salt medium. The biosynthesis of HA was elicited by tomato tissue in PDB (both full and 1/5 strength). On the contrary, this was not observed for iso HA, whose accumulation was reduced by the presence of plant tissue added in the cultivation substrate. However no significant differences were observed in salt medium amended or not with tomato tissue (data not shown). Moreover, HA and iso-HA were not detected in the mycelium extracts (data not shown).

**Figure 8:**
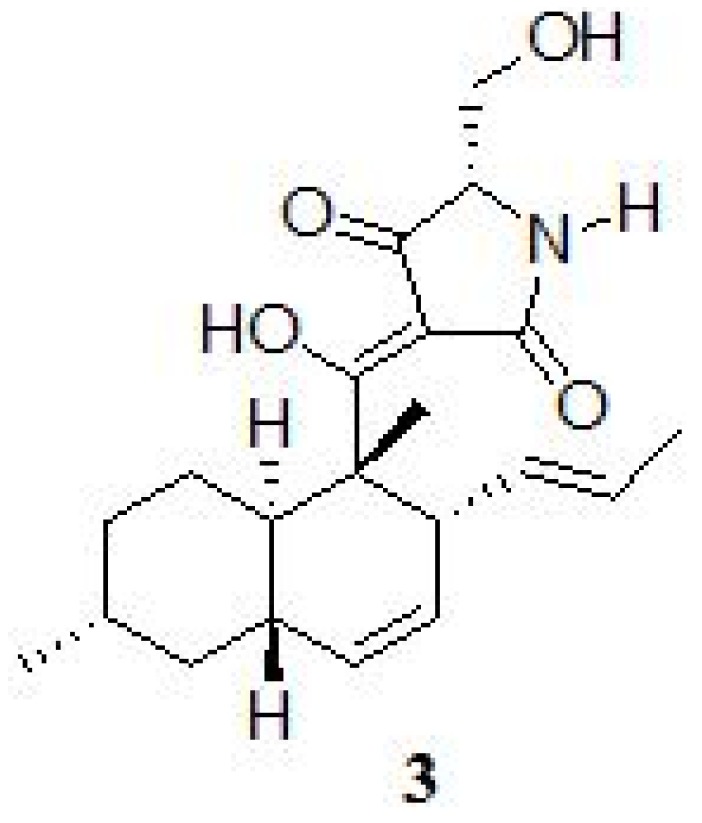
Chemical structure of trichosedin (**3**).

Interestingly, the dual culture of *T. harzianum* and calli of *Catharathus roseus* produced another tetramic acid compound named trichosedin (**3 ** in Figure 8), that was not produced in the single culture of *T. harzianum* or *C. roseus* callus [18]. This fungal metabolite affects the root and shoot growth of several plant species [19]. Dual cultures of a fungus and a plant provide a simple method of establishing plant-fungus interaction and allow isolation of metabolites induced by one of the system components. Previous studies also demonstrated that the production of secondary metabolites was induced by fungal cell wall material or by the presence of pathogens [11].

## 3. Experimental Section

### 3.1. General Information

Chromatographic separation was performed using an HPLC (1260 Infinity; Agilent Technologies, Santa Clara, CA, USA) equipped with a Diode Array Detector (DAD) and a Zorbax Eclipse Plus C18 3.5 μm column (100 × 4.6 mm—Agilent). The eluents were: **A** = water + 0.1% formic acid, and **B** = acetonitrile. The gradient program was as follows: 10%–100% B (18 min), 100% B (16 min) at a constant flow of 0.5 mL min^−1^ (injection volume: 20 µL). A Prodigy 10 μm ODS column (250 × 10 mm, Phenomenex Torrance, CA, USA) was used for semi-preparative HPLC (gradient program as reported above at a constant flow of 3 mL min^−1^, injection volume: 500 µL). ^1^H and ^13^C-NMR spectra were recorded with a Bruker Avance 400 instrument (Bruker Biospin, Rheinstetten, Germany) operating at 400 (^1^H) and 100 (^13^C) MHz, using residual and deuterated solvent peaks as reference standards. Electrospray mass spectra were recorded on a Agilent LCMS QTOF 6540 (Agilent Technologies, Santa Clara, CA, USA).

### 3.2. Fungal Strains

The phytopathogens *R. solani*, *S. sclerotiorum*, *P. ultimum* and *Botrytis cinerea*, as well as the antagonistic fungus, *T. harzianum* strain M10 were maintained on potato dextrose agar (PDA, Sigma, St Louis, MO, USA) at room temperature and sub-cultured bimonthly. Two 7-mm diameter plugs of *T. harzianum*, obtained from actively growing margins of PDA cultures, were inoculated into conical flasks containing 1.5 L of sterile potato dextrose broth (PDB). The stationary cultures were incubated for 30 days at 25 °C. 

### 3.3. Production and Isolation of Trichoderma Secondary Metabolites

The cultures of *T. harzianum* strain M10 were filtered under vacuum through filter paper (No. 4, Whatman, Brentford, UK), and the filtrates stored at 2 °C for 24 h. The filtered culture broth (2 L) was acidified to pH 4 with 5 M HCl and extracted exhaustively with ethyl acetate (EtOAc). The combined organic fraction was dried (Na_2_SO_4_) and evaporated *in vacuo* at 35 °C. The red residue recovered was dissolved in CHCl_3_ and extracted three times with 2 M NaOH. Harzianic acid (HA) and isoharzianic acid (iso-HA) were then precipitated with 2 M HCl. The solid phase was recovered (135 mg), solubilised and subjected to RP-18 vacuum chromatography (20 g), eluting with a gradient of methanol (MeOH):H_2_O:CH_3_CN (1:8:1 to 10:0:0), or semi-preparative HPLC (see “Production of HA and iso-HA in the presence of plant tissue” section). After the separation, 45 mg of pure HA and 30 mg of iso-HA were collected. The homogeneity of pure pooled products was verified by analytical reverse-phase TLC (glass pre-coated Silica gel 60 RP-18 plates—Merck Kieselgel 60 TLC Silica gel 60 RP-18 F254s, 0.25 mm) using 3:4:3 CH_3_CN:MeOH:H_2_O as eluent (Rf of HA: 0.3). The compounds were detected on TLC plates using UV light (254 or 366 nm) and/or by spraying the plates with 5% (*v/v*) H_2_SO_4_ in EtOH followed by heating at 110 °C for 10 min. NMR data: see Table 1. LC-MS fragmentation patterns of iso-HA and HA: HA: [M+H]^+^ = 366.2; [M+H-H_2_O]^+^ = 348.2; [M+H-HCO_2_H]^+^ = 320.2; [M+H-HCO_2_H-CH_3_-CH_2_-CH_2_-CH=CH-CH=CH_2_]^+^ = 224.1; [M+2H-HCO_2_H-(CH_3_-CH_2_-CH_2_-CH=CH-CH=CH-CH-OH)-(CH_3_-CH-CH_3_)-CH_3_]^+^ = 138.0; iso-HA [M+H]^+^ = 366.2; [M+H-HCO_2_H]^+^ = 320.2; [M+H-HCO_2_H-CH_3_-CH_2_-CH_2_-CH=CH-CH=CH_2_]^+^ = 224.1; [M+H-HCO_2_H-CH_3_-CH_2_-CH_2_-CH=CH-CH=CH_2_]^+^ = 224.1; [M+2H-HCO_2_H-(CH_3_-CH_2_-CH_2_-CH=CH-CH=CH-CH-OH)-(CH_3_-CH-CH_3_)-CH_3_]^+^ = 138.0.

### 3.4. Antifungal Assay

Iso HA was tested against *R. solani*, *S. sclerotiorum*, *P. ultimum* and *B. cinerea* to evaluate its antifungal properties. Pathogen plugs (5-mm diameter) from growing edges of colonies were placed at the centre of Petri dishes containing PDA. Iso HA was assayed starting from a 10^−2^ M water solution. The pathogen growth was measured daily as colony diameter for ten days. Each treatment consisted of three replicates and the experiment was repeated twice.

### 3.5. In Vitro Plant Growth Promotion

Seeds of tomato (*Solanum lycopersicum* cv. San Marzano) were sterilized with 1% sodium hypochlorite for 5 min, then rinsed 3 times with sterile distilled water. The seeds were allowed to germinate in Petri dishes (150 mm diameter) containing Murashighe & Skoog (MS) culture medium, 1% agar and 1% sucrose. HA and iso HA water dilutions (10^−5^, 10^−6^, 10^−7^, 10^−8^ and 10^−9^ M) were added separately into the substrate. Six tomato seeds were placed in each Petri dish and three replicates were tested for each salt medium concentration. After germination, the plates were incubated in a growth chamber (25 °C, 16 h of photoperiod). The effect of the treatments was evaluated by measuring the percent of seed germination and the length of roots and stems every 24 h for 7 days. The experiment was repeated three times.

### 3.6. Plant Growth Promotion and Induction of Resistance

Fifteen days-old tomato seedlings cv. San Marzano were transplanted in 14 cm pots containing sterilized soil and incubated in a growth chamber (25 °C, 16 h photoperiod). Each plant was treated, on alternate days, by drenching with aqueous solutions of iso HA at three different concentrations (10^−5^, 10^−6^, and 10^−7^ M). Each treatment consisted of five replicates and water treated plants were used as control. The plant growth was measured after four weeks in terms of stem length. Furthermore the metabolite ability to induce systemic disease resistance against *B. cinerea* was determined. Three leaves of each stage were infected with 10 μL of a pathogen spore suspension (1 × 10^5^ spore/mL) in germination buffer (PDB 1/5 strength). The incidence of the disease was evaluated by measuring, up to 72 h post-infection, the necrotic area (mm^2^) on infected leaves as compared to water-treated samples. The experiment was repeated twice and data were combined since statistical analysis determined homogeneity of variance (*p* ≤ 0.05).

### 3.7. Production of HA and Iso HA in Presence of Plant Tissue

The production of HA and iso HA by *T. harzianum* in liquid cultures amended with plant tissue was evaluated. Tomato seedlings (*Solanum lycopersicum* cv. San Marzano), 15 day-old, were harvested, surface sterilised with sodium hypochlorite 1% solution, homogenized and added to the substrate (PDB, 1/5 strenght PDB or salt medium +1% glucose) at a concentration of 10 g L^−1^. Four 7 mm diameter plugs of *T. harzianum*, obtained from actively growing margins of PDA cultures, were inoculated to 500 mL conical flasks containing 100 mL of medium. Culture broths of *T. harzianum* without amendment with plant were used as controls. The stationary cultures were incubated for 10, 20, 30 and 40 days at 25 °C and then filtered under vacuum. The filtered broths (0.2 μm) were subjected to HPLC/DAD analysis for the quantification of metabolites. Each treatment consisted of four replicates and the experiment was repeated twice. Data from the experiments were combined since statistical analysis determined homogeneity of variance (*p* ≤ 0.05).

## 4. Conclusions

The results reported in this work indicated that HA and the new secondary metabolite iso HA are involved in *Trichoderma*—plant interactions and can be useful for agricultural and non-agricultural applications [20]. The identification of molecules with such biological activities can support the development of new biopesticides and biofertilisers based on fungal compounds as active ingredients, which represent an attractive alternative to products containing only living microorganisms.
